# Efficacy of Vitamin D Supplementation to Alleviate Premenstrual Syndrome Symptoms: A Systematic Review and Meta-Analysis of Randomized Controlled Trials

**DOI:** 10.3390/jcm15124828

**Published:** 2026-06-22

**Authors:** Amani Zainab, Reem Samir Tageldin, Rumaysah Patel, Mohamed Abdulrahim Hassan, Lama Atef Aburas, Marya Alkahily, Layan Shamsan, Salma Salman Al-Olaimat, Ashaz Sayeed, Ahmed Abu-Zaid

**Affiliations:** 1 College of Medicine, Alfaisal University, Riyadh 11533, Saudi Arabia; 2 Department of Biochemistry and Molecular Medicine, College of Medicine, Alfaisal University, Riyadh 11533, Saudi Arabia

**Keywords:** vitamin D, premenstrual syndrome, premenstrual dysphoric disorder, pain, meta-analysis

## Abstract

**Aim**: Premenstrual Syndrome (PMS) substantially impairs quality of life, while standard pharmacologic treatments are often limited by adverse effects or contraindications. This systematic review and meta-analysis evaluated the efficacy of vitamin D supplementation compared to passive control (placebo or standard care) for PMS symptom relief. **Methods**: PubMed, Web of Science, Scopus, Google Scholar, and CENTRAL were searched for randomized controlled trials (RCTs) up to January 2026. The primary outcome was change in total PMS severity scores. Secondary outcomes included depression, anxiety, physical symptoms, craving, and water retention. Standardized mean differences (SMDs) with 95% confidence intervals (CI) were pooled using a random-effects model. Heterogeneity was assessed using the I^2^ statistic. **Results**: Five RCTs including 436 participants were reviewed. The overall risk of bias was rated as “some concerns” in four RCTs and “low risk” in one trial. Vitamin D significantly reduced total PMS severity (SMD: −0.78, 95% CI: −1.30, −0.26, I^2^ = 58.22%) as well as physical symptoms (SMD: −1.00, 95% CI: −1.99, −0.01, I^2^ = 90.53%) and depression (SMD: −0.78, 95% CI: −1.53, −0.02, I^2^ = 84.56%). No significant effects were observed for anxiety, craving, or water retention. Vitamin D was well tolerated with no reported adverse events. **Conclusions**: Vitamin D supplementation may reduce overall PMS severity, particularly physical and depressive symptoms. However, results show substantial heterogeneity across outcomes, and the certainty of evidence remains “very low”, underscoring the need for further high-quality, well-powered RCTs.

## 1. Introduction

Premenstrual Syndrome (PMS) is a multifaceted condition marked by the recurrent onset of irritating behavioral, psychological, and physical symptoms that recur during the luteal phase of the menstrual cycle, which wanes shortly after menstruation begins [1]. Epidemiological data indicate that up to 90% of women of reproductive age are affected by PMS, with 20–40% experiencing symptoms severe enough to necessitate medical intervention [2,3]. PMS considerably impacts quality of life, often disrupting social functioning and personal relationships [4]. Additionally, the economic consequences are significant, resulting from decreased occupational productivity, increased absenteeism, and presenteeism [5].

Although the causes of PMS are not entirely clear, it is associated with an atypical sensitivity to regular cyclical hormonal shifts [6]. These hormonal fluctuations affect central neurotransmitters, disrupting gamma-aminobutyric acid (GABA) as well as serotonin pathways [7]. Dysregulation of the hypothalamic–pituitary–adrenal (HPA) axis has also been implicated in PMS pathophysiology. Altered stress responsiveness and cortisol secretion patterns may contribute to mood instability and increased emotional reactivity during the luteal phase [8]. Alterations in prolactin secretion have been investigated in PMS, with some early hypotheses suggesting a possible association with symptoms such as breast tenderness and mood changes [9]. However, overall evidence does not support a consistent or clinically meaningful role for prolactin in the pathophysiology of PMS [10,11]. Genetic susceptibility is another important factor in PMS development. Familial clustering of symptoms suggests a potential heritable component, with candidate gene studies implicating polymorphisms in serotonin [12] and estrogen [13] receptors. These genetic variations may influence individual sensitivity to hormonal fluctuations and neurotransmitter regulation across the menstrual cycle.

Standard first-line management currently includes lifestyle modifications, selective serotonin reuptake inhibitors, and oral contraceptives [7,14,15]. Despite their efficacy, pharmacological interventions are often limited by low adherence due to adverse effects such as nausea, fatigue, decreased libido, and weight gain [14,15]. Additionally, these options may be inadvisable for women seeking pregnancy, which results in the continuous necessity for effective, safe, and natural therapeutic options. Other commonly used therapeutic options include nonsteroidal anti-inflammatory drugs (NSAIDs) for premenstrual and menstrual physical symptoms [16,17], calcium supplementation [18], and in selected cases diuretics such as spironolactone for mood-, somatic-, and fluid-related symptoms [19,20]. Cognitive behavioral therapy and structured exercise programs have also shown some potential benefit in reducing symptoms, although the current evidence base remains limited [21]. For severe or refractory cases, gonadotropin-releasing hormone (GnRH) agonists are beneficial and may be considered under specialist supervision [22].

Vitamin D has been identified as a potential therapeutic agent owing to its function as a key neurosteroid [23]. The widespread distribution of vitamin D receptors throughout brain regions that control mood and behavior, such as the hippocampus and hypothalamus, supports the biological plausibility of its efficacy in PMS [24]. Vitamin D may mitigate PMS symptoms by increasing tryptophan hydroxylase 2 expression, the rate-limiting enzyme in serotonin production [25]. Additionally, it significantly regulates intracellular calcium homeostasis and shows anti-inflammatory effects that may alleviate physical symptoms such as cramping and pain [26]. These mechanisms are supported by observational research showing an inverse relationship between serum 25(OH)D concentrations and the severity of premenstrual symptoms [27].

Despite this strong biological rationale, results from randomized controlled trials (RCTs) examining the therapeutic benefits of vitamin D supplementation for PMS remain inconsistent [28,29,30,31,32]. While some trials report significant improvements in both physical and psychological domains [29,30], others have found no significant benefit over placebo or conflicting results regarding specific symptom domains [31,32]. Thus, this research seeks to assess the efficacy of vitamin D supplementation in alleviating both the psychological as well as psychological symptoms of PMS.

## 2. Methods

### 2.1. Study Protocol

The study design and reporting adhered strictly to the PRISMA (Preferred Reporting Items for Systematic Reviews and Meta-Analyses) guidelines [33] and followed the methods outlined in the Cochrane Handbook for Systematic Reviews of Interventions [34]. The review was not prospectively registered. The PRISMA checklist is depicted in Table S1.

### 2.2. Databases and Strategy of Search

A literature screen was executed on 10 January 2026, across several data sources, including Web of Science, PubMed, Google Scholar, CENTRAL, and Scopus. We formed a search strategy, utilizing the following string: (“Vitamin D” OR “Cholecalciferol” OR “Vitamin D3” OR “25-hydroxyvitamin D”) AND (“Premenstrual Syndrome” OR PMS OR “Premenstrual Tension” OR PMDD OR “Premenstrual Dysphoric Disorder”). No filters were used. In addition, the reference lists of all included studies were hand-searched to detect any eligible studies that may have been missed by the electronic search strategy. Comprehensive search strategies along with the corresponding results for each database are depicted in Table S2.

### 2.3. Eligibility Criteria

We considered RCTs that met predefined PICO criteria. The population consisted of adolescent and adult women diagnosed with PMS or premenstrual dysphoric disorder (PMDD) using validated diagnostic tools such as the Premenstrual Symptoms Screening Tool (PSST), regardless of vitamin D deficiency status. The intervention involved vitamin D supplementation administered via any route, including oral or parenteral, at any dosage or frequency. The control group received placebo, no treatment, or standard care such as lifestyle education. The primary outcome was the change in total PMS symptom severity scores, while secondary outcomes included changes in specific symptom domains (depressive symptoms, anxiety, physical/somatic symptoms, craving, and water retention) as well as the incidence of adverse events. Studies reporting at least one of the prespecified primary or secondary outcomes were regarded eligible for inclusion.

### 2.4. Study Selection

All identified records were imported into Covidence for automatic duplicate removal. Next, two independent reviewers evaluated the titles and abstracts for relevance. Potentially eligible studies underwent a full-text review against the inclusion criteria. Any disagreements regarding study selection were resolved by discussion, and when necessary, through adjudication by a third senior investigator.

### 2.5. Data Extraction

Data collection was completed independently by two groups, each consisting of two investigators, using a pre-piloted Excel-based form, with any discrepancies resolved through consultation with a third senior investigator. Collected variables comprised study characteristics (first author, year of publication, country, study design, sample size, intervention and control details, vitamin D status, inclusion conditions, primary outcomes, and follow-up period), participant characteristics (age, body mass index, baseline serum vitamin D levels, and baseline PMS severity scores), and outcome data (mean and standard deviation for continuous outcomes such as PMS scores and symptom domains, and event counts for dichotomous outcomes such as adverse events). Where data were reported as medians and interquartile ranges, these were transformed to means and standard deviations utilizing the technique designed by Wan et al. [35].

### 2.6. Quality Assessment and Certainty of Evidence

The risk of bias for individual RCTs was assessed independently by at least two investigators using the Cochrane Risk of Bias 2 (RoB 2) tool as per protocol [36]. Additionally, the certainty of the pooled evidence was evaluated using the GRADE (Grading of Recommendations Assessment, Development, and Evaluation) approach as per protocol [37,38]. Disagreements were rectified through consensus.

### 2.7. Meta-Analysis

Statistical computations were conducted using Stata/SE software, version 19 (StataCorp, College Station, TX, USA). Continuous outcomes were analyzed using standardized mean differences (SMDs) with 95% confidence intervals (CIs). The SMD was used to account for differences in the scales used to assess PMS symptom severity across studies. A random-effects model (REML) was applied for all outcomes to account for observed heterogeneity in treatment protocols. Heterogeneity was assessed using the chi-squared test and the I^2^ statistic, with *p* < 0.10 and I^2^ > 50% indicating significant heterogeneity. Sources of heterogeneity were explored through sensitivity analyses using the leave-one-out method and Galbraith plots. Publication bias was not assessed, as fewer than 10 studies were available for each outcome [39]. Statistical significance was set at a two-sided *p*-value of <0.05 for all analyses.

## 3. Results

### 3.1. Summary of Search Results and Study Selection

**Figure 1:**
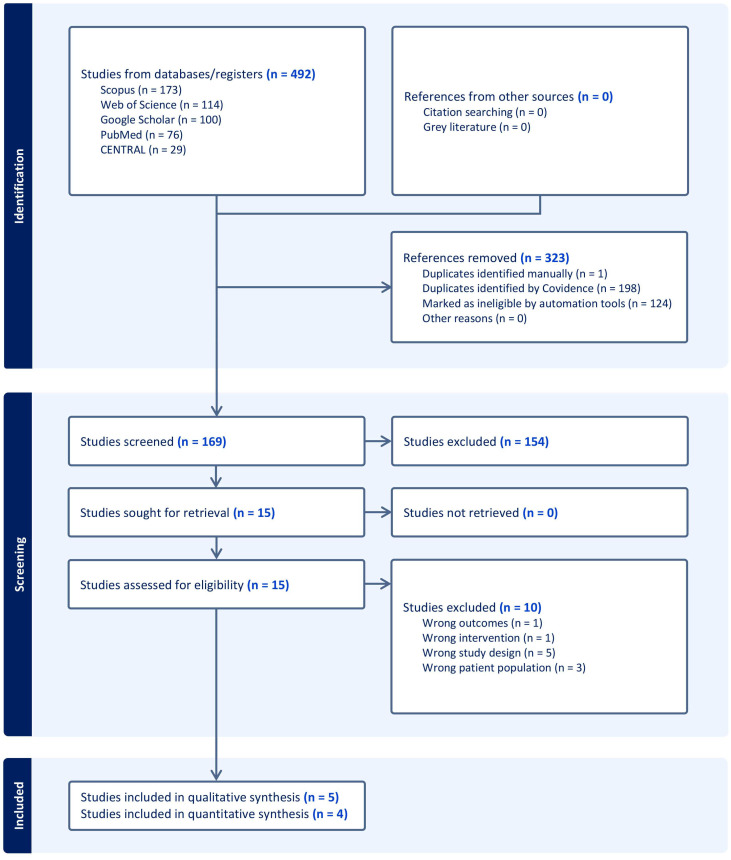
PRISMA flow chart of the screening process.

Overall, 492 records were detected through database searching. Following the removal of 323 duplicate and ineligible records, 169 records remained for title and abstract screening. During the screening process, 154 records were excluded. Then, 15 full texts were assessed for eligibility. Of these, 10 studies were excluded for different reasons (Table S3). Finally, five studies met the inclusion criteria and were included in the systematic review [28,29,30,31,32], Of these, four studies [29,30,31,32] were included in the meta-analysis, while one study [28] was not quantitatively pooled because it did not provide extractable data for the meta-analyzed outcomes (Figure 1).

### 3.2. Summary of the Characteristics of the Included Studies

Five RCTs involving a total of 436 patients were included in our synthesis [28,29,30,31,32]. The studies were primarily conducted in Iran, with one study also performed in Italy. The duration of the intervention varied from 2 to 4 months, and vitamin D dosing regimens differed across studies. Further details of the study designs are presented in Table 1, while baseline characteristics of the included participants are summarized in Table 2.

### 3.3. Summary of the Risk of Bias and Certainty of Evidence

**Figure 2:**
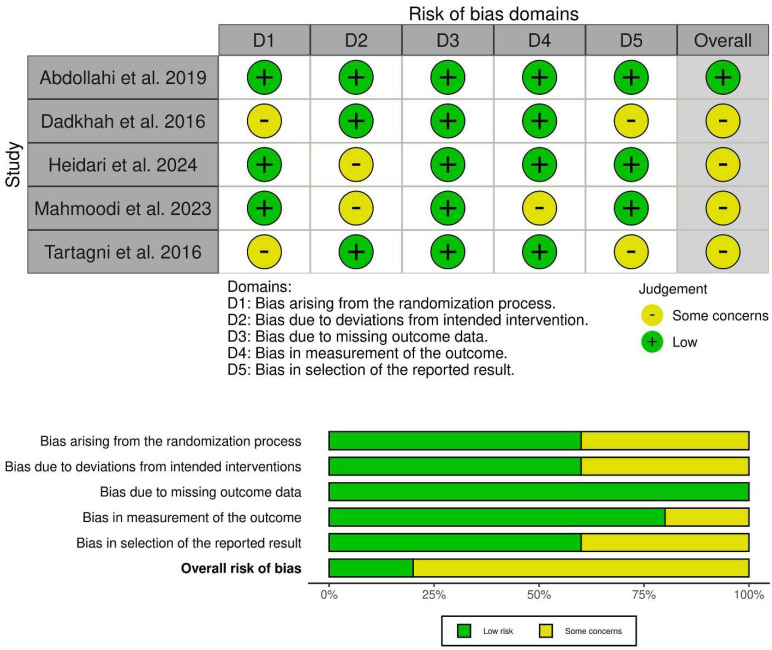
Quality assessment of risk of bias in the included trials [28,29,30,31,32].

One RCT showed a low risk of bias overall [32], and four trials showed some concerns overall [28,29,30,31] (Figure 2). Two trials raised concerns about selection bias because details of the randomization process were absent [28,31]. The same two trials raised concerns about reporting bias because they lacked a prospectively registered protocol [28,31]. Two trials raised concerns about performance bias due to open-label interventions [29] or per-protocol analysis [30].

The certainty of the evidence was rated as “very low” for all analyzed outcomes, indicating that the findings were weak and highly uncertain; therefore, any conclusions drawn from them should be interpreted with caution (Table 3).

### 3.4. Primary Outcome: PMS Total Score

**Figure 3:**
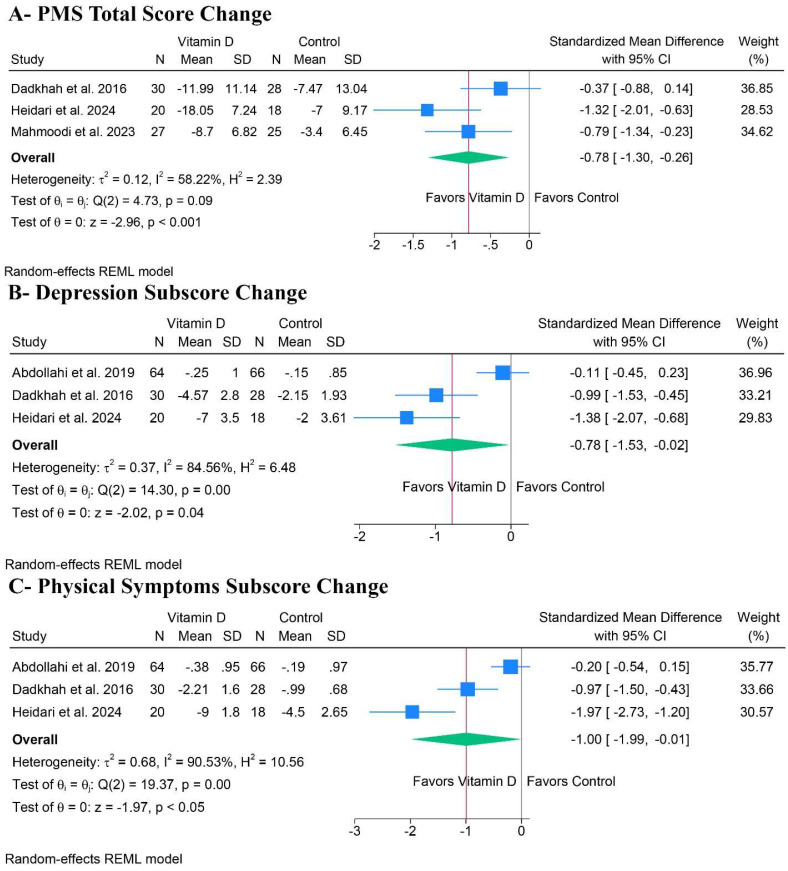
Forest plots of PMS total score change (**A**), depression subscore change (**B**), and physical symptoms subscore change (**C**) [29,30,31,32].

Vitamin D supplementation was linked to a significantly greater reduction in total PMS scores contrasted to the control group (n = 3 RCTs; SMD: −0.78; 95% CI [−1.30, −0.26]; *p* < 0.001; I^2^ = 58.22%) (Figure 3A). A leave-one-out sensitivity analysis of the total PMS score depicted that the result became statistically non-significant after omitting Mahmoodi et al. (n = 2 RCTs, SMD: −0.81, 95% CI [−1.74, 0.11], *p* = 0.09) (Figure S1). The Galbraith plot revealed no potential outliers (Figure S2).

### 3.5. Secondary Sub-Scores

**Figure 4:**
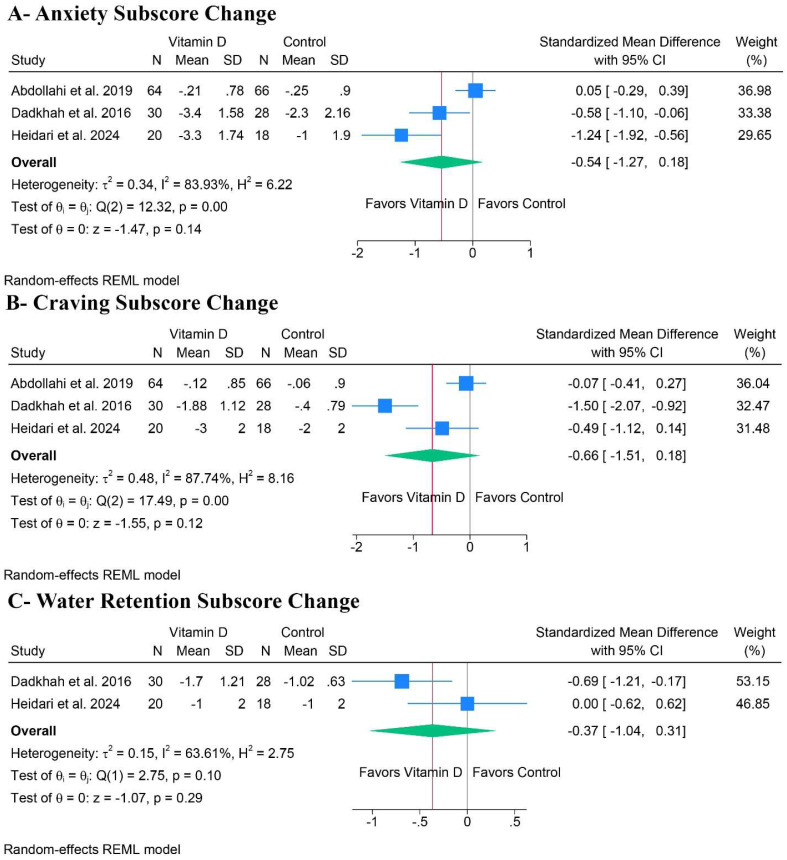
Forest plots of anxiety subscore change (**A**), craving subscore change (**B**), and water retention subscore change (**C**); CI: confidence interval [30,31,32].

Vitamin D supplementation was linked to a significantly greater reduction in depression sub-score (n = 3 RCTs; SMD: −0.78; 95% CI [−1.53, −0.02]; *p* = 0.04; I^2^ = 84.56%) (Figure 3B) and physical symptoms sub-score (n = 3 RCTs; SMD: −1.00; 95% CI [−1.99, −0.01]; *p* < 0.05; I^2^ = 90.53%) (Figure 3C). However, no significant change was noted between both groups regarding anxiety sub-score (n = 3 RCTs; SMD: −0.54; 95% CI [−1.27, 0.18]; *p* = 0.14; I^2^ = 83.93%) (Figure 4A), craving sub-score (n = 3 RCTs; SMD: −0.66; 95% CI [−1.51, 0.18]; *p* = 0.12; I^2^ = 87.74%) (Figure 4B), and water retention sub-score (n = 2 RCTs; SMD: −0.37; 95% CI [−1.04, 0.31]; *p* = 0.29; I^2^ = 63.61%) (Figure 4C).

For the depression and physical symptom sub-scores, the results were robust to the exclusion of Abdollahi et al. [32], but lost statistical significance when omitting Dadkhah et al. [31] or Heidari et al. [30] (Figures S3 and S4). Also, the Galbraith plots identified Heidari et al. [30] and Dadkhah et al. [31] as potential outliers contributing to the observed heterogeneity across all outcomes (Figures S5 and S6).

Additionally, a leave-one-out sensitivity analysis of anxiety sub-score revealed that the result became statistically significant after omitting Abdollahi et al. [32] (n = 2 RCTs; SMD: −0.87; 95% CI [−1.51, −0.22]; *p* = 0.008) (Figure S7). Also, the Galbraith plot identified Heidari et al. [30] and Dadkhah et al. [31] as potential outliers contributing to the observed heterogeneity (Figure S8).

Regarding craving scores, the sensitivity analysis indicated that the result became statistically significant after omitting Abdollahi et al. (n = 2 RCTs; SMD: −1; 95% CI [−1.99, −0.01]; *p* = 0.047) (Figure S9). Also, the Galbraith plot identified Dadkhah et al. as a potential outlier contributing to the observed heterogeneity (Figure S10).

### 3.6. Tolerability and Patient Satisfaction

Regarding safety, vitamin D supplementation was well tolerated across the included trials, as none reported adverse events in patients receiving the intervention [28,29,30,31,32]. Additionally, Mahmoodi et al. evaluated participant satisfaction, reporting that the vitamin D group had higher satisfaction (51.4 ± 12.0), while the online lifestyle modification group reported lower satisfaction (43.0 ± 3.3) [29]. Within-group analysis indicated that satisfaction scores improved significantly between Week 6 and Week 10 only in the vitamin D group (*p* = 0.04), with no substantial difference noted in the control group (*p* = 0.622) [29].

## 4. Discussion

### 4.1. Summary of Principal Findings

The synthesis of data from the five RCTs and 436 participants revealed that vitamin D supplementation was linked to a statistically significant decrease in total PMS symptom severity scores compared to the control group. However, the efficacy profile was not stable across the symptom domains of PMS. Vitamin D significantly alleviated physical symptoms and depression; still, there was no difference in the domains of anxiety, craving, and water retention. Additionally, the certainty of this evidence remained “very low” according to GRADE criteria, primarily due to serious risk of bias, inconsistency, and imprecision.

### 4.2. Interpretation of Findings

The most tangible efficacy signal was observed for physical symptoms, including abdominal cramping, mastalgia, back pain, and generalized muscle aches. This finding can be biologically plausible given the calcium-regulation hypothesis of PMS [40,41]. Extracellular calcium deficiency is associated with a reduced neuromuscular excitation threshold, leading to hyperexcitability as evidenced by spontaneous motor unit firing, muscle cramping, and smooth muscle contraction [40]. Insufficient vitamin D can lead to decreased serum ionized calcium levels or secondary hyperparathyroidism, which maintains serum levels by depleting intracellular stores [42]. In women with PMS, altered patterns of calcium-regulating hormones and calcium/vitamin D intake have been implicated in symptom severity. Still, the specific interactions among ovarian steroid fluctuations, calcium kinetics, and PTH regulation remain incompletely defined [43,44].

One possible explanation is that cyclical hormonal changes may interact with marginal vitamin D status and calcium homeostasis, increasing susceptibility to neuromuscular symptoms in some women [40,41]. Accordingly, vitamin D supplementation could, in theory, improve calcium absorption and neuromuscular stability, thereby reducing cramping or musculoskeletal discomfort. However, this mechanism was not directly tested in the included trials and should be considered hypothesis-generating. Thus, restoring normal calcium dynamics may provide a central analgesic effect, along with its peripheral impact on muscles, suggesting a dual mechanism for reducing pain-related physical symptoms.

Moreover, the reduction in PMS psychological symptoms, even if not statistically significant in some domains, correlates with the developing understanding of vitamin D as a pleiotropic neurosteroid, beyond its function as a regulator of calcium homeostasis. Vitamin D receptors and the 1α-hydroxylase enzyme are highly expressed in the hippocampus, hypothalamus, and substantia nigra, which are key regions for emotional regulation and reward processing [23,45]. The active metabolite of vitamin D (1,25-dihydroxyvitamin D) regulates the production of tryptophan hydroxylase 2, which is the rate-limiting enzyme of serotonin production [25,46]. Serotonergic dysregulation has been implicated in PMS-related mood and appetite symptoms, and vitamin D modulation of serotonin synthesis has been proposed as a potential mechanism [47,48]. However, direct evidence that vitamin D improves PMS by modifying luteal-phase serotonergic activity remains lacking.

However, the lack of a significant effect on water retention should be interpreted cautiously, as this outcome was pooled from only two trials (n = 96 participants), and is therefore underpowered. Although vitamin D has been described as a negative regulator of the renin–angiotensin system, it remains unclear whether this pathway is clinically relevant to PMS-related edema or fluid retention [49,50]. Water retention in PMS is likely multifactorial, and available trial data are insufficient to determine whether vitamin D has a meaningful effect on this domain.

Notably, sensitivity analyses indicated that the non-significant pooled effects for anxiety and craving were driven primarily by the inclusion of Abdollahi et al. [32]. Exclusion of this study resulted in statistically significant effects, suggesting that this trial had a substantial influence on the overall pooled estimates and heterogeneity.

### 4.3. Strengths and Limitations

Despite our review presenting the most comprehensive synthesis to date on the efficacy of vitamin D in alleviating PMS symptoms, our results have several limitations. First, the findings are limited by significant statistical heterogeneity, with I^2^ values >75% for depression (84.56%) and physical symptoms (90.53%), representing “considerable” heterogeneity and substantially weakening confidence in the pooled estimates, which may reflect differences in treatment protocols. The included studies utilized different vitamin D regimens, ranging from daily lower doses in Dadkhah et al. [31] to high-dose bolus administration in Tartagni et al. [28] and Mahmoodi et al. [29]. Second, most included trials were conducted in Iran, which significantly limits the generalizability of our findings. Also, the small sample sizes in some outcomes, such as water retention, further limit the robustness of our conclusions. Third, eligibility criteria differed across trials regarding baseline vitamin D status. This may have diluted the apparent treatment effect if supplementation is primarily beneficial in women with deficiency or insufficiency, while offering limited additional benefit in vitamin D-replete participants. In addition, baseline PMS severity scores and vitamin D levels were each unreported in two trials, limiting full assessment of baseline comparability in symptom severity and vitamin D status across studies. Finally, the “very low” certainty of evidence due to the previously mentioned limitations further limits the applicability of our results before more robust confirmation.

### 4.4. Clinical Implications and Future Directions

Clinically, vitamin D may be a promising, low-cost, and accessible adjunct therapy for women with PMS, particularly those with vitamin D insufficiency. However, it should not be considered a replacement for established first-line treatments, including lifestyle interventions, cognitive behavioral therapy, and pharmacological options such as SSRIs and oral contraceptives, which remain the cornerstone of management for mild to moderate and severe cases [7,14,15]. Vitamin D may offer a supplementary option for patients who are hesitant to use SSRIs due to side effects or stigma [15]. Overall, existing evidence remains inadequate and unconcreted, and any clinical application should be attempted with caution pending additional high-quality RCTs.

For future research, prospective trials should adopt standardized dosing protocols to facilitate comparable synthesis and treatment recommendations. Also, the efficacy of vitamin D may depend on baseline serum levels; future RCTs should stratify participants by vitamin D status (insufficiency/deficiency vs. sufficiency) to determine whether the clinical benefit is restricted to repleting a deficiency or whether supra-therapeutic doses provide additional pharmacological benefit.

## 5. Conclusions

Vitamin D supplementation may offer symptom alleviation in patients with PMS, especially for physical symptoms and depression. However, there was no difference between vitamin D treatment and control regarding anxiety, craving, and water retention. Additionally, the current evidence remains scarce and uncertain, with a limited heterogeneous number of RCTs, meriting additional large-sized RCTs prior to clinical advocacy.

## Figures and Tables

**Table 1:** Summary characteristics of the included RCTs.

Study ID	Country	n	Vitamin D Group Details	Control Group Details	Vitamin D Status	Main Inclusion Criteria	Primary Outcome	Follow-Up Duration
Abdollahi et al. 2019 [32]	Iran	130	Vitamin D (2000 IU) tablet every other day for 3 months	Placebo tablet every other day	Deficient (<20 ng/mL)	Age 18–30; Single; PMS diagnosis (PSST)	PMS symptoms (PSST); Serum 25(OH)D	3 months
Dadkhah et al. 2016 [31]	Iran	58	Vitamin D tablet (200 mg) daily for 2 months	Placebo tablet daily	Not specified	Age 15–45; PMS diagnosis; Regular menstrual cycles	PMS symptom severity (daily symptom record)	2 months
Heidari et al. 2024 [30]	Iran	38	Vitamin D (50,000 IU) pearl fortnightly (every 2 weeks) for 4 months	Placebo pearl containing edible paraffin fortnightly	Insufficient (21–29 ng/mL)	Age 18–25; Single; PMS diagnosis	Inflammatory markers (IL-10, IL-12, TAC); PMS symptom severity	4 months
Mahmoodi et al. 2023 [29]	Iran	52	Vitamin D3 (50,000 IU) tablet weekly for 6 weeks	Online lifestyle training (General education on diet, exercise, and stress)	Deficient/Insufficient (≤30 ng/mL)	Age 20–40; PMS diagnosis (PSST)	PMS symptoms (PSST)	10 Weeks
Tartagni et al. 2016 [28]	Italy	158	High-dose Vitamin D (200,000 IU initial dose, then 25,000 IU every 2 weeks) for 4 months	Placebo	Severe deficiency (≤10 ng/mL)	Age 15–21; PMS-related mood disorders	Emotional and cognitive symptoms (anxiety, irritability, sadness)	4 months

25(OH)D: 25-hydroxyvitamin D; IL: Interleukin; IU: International Units; PMS: Premenstrual Syndrome; PSST: Premenstrual Symptoms Screening Tool; RCT: Randomized Controlled Trial; TAC: Total Antioxidant Capacity.

**Table 2:** Baseline characteristics of the participants.

Study ID	Sample Size (n)		Age (Years) Mean ± SD		BMI (kg/m^2^) Mean ± SD		Baseline Vitamin D Level Mean ± SD		Baseline PMS Score Mean ± SD	
	Vitamin D	Control	Vitamin D	Control	Vitamin D	Control	Vitamin D	Control	Vitamin D	Control
Abdollahi et al. 2019 [32]	64	66	22.5 ± 2.6	22.7 ± 3.0	21.4 ± 2.6	21.7 ± 3.3	5.4 ± 5.7	4.9 ± 4.6	NR	NR
Dadkhah et al. 2016 [31]	30	28	30.9 ± 6.9	29.6 ± 5.5	23.7 ± 5.8	23.6 ± 3.6	NR	NR	37.49 ± 10.72	35.21 ± 13.62
Heidari et al. 2024 [30]	20	18	21.3 ± 1.6	21.7 ± 1.8	20.7 ± 1.2	21.4 ± 2.0	21.4 ± 7.6	21.1 ± 6.8	39 ± 8	35 ± 10
Mahmoodi et al. 2023 [29]	27	25	31.9 ± 5.6	33.0 ± 3.6	NR	NR	16.9 ± 4.1	Not Measured	34.1 ± 7.1	35.2 ± 6.4
Tartagni et al. 2016 [28]	80	78	19.8 ± 1.4	18.6 ± 1.9	22.1 ± 1.4	22.5 ± 1.1	NR	NR	NR	NR

BMI: Body Mass Index; NR: Not Reported; PMS: Premenstrual Syndrome; SD: Standard Deviation.

**Table 3:** GRADE evidence profile.

Certainty Assessment							Effect Estimate with Vitamin D
Participants (Studies) Follow-Up	Risk of Bias	Inconsistency	Indirectness	Imprecision	Publication Bias	Overall Certainty of Evidence	
Total PMS Score							
148 (3 RCTs)	serious ^a^	serious ^b^	not serious	very serious ^c^	none	⨁◯◯◯ Very low ^a,b,c^	SMD 0.78 SD lower (1.3 lower to 0.26 lower)
Depression Sub-score							
226 (3 RCTs)	serious ^a^	very serious ^d^	not serious	very serious ^c^	none	⨁◯◯◯ Very low ^a,c,d^	SMD 0.78 SD lower (1.53 lower to 0.02 lower)
Physical Symptoms Sub-score							
226 (3 RCTs)	serious ^a^	very serious ^d^	not serious	very serious ^c^	none	⨁◯◯◯ Very low ^a,c,d^	SMD 1 SD lower (1.99 lower to 0.01 lower)
Anxiety Sub-score							
226 (3 RCTs)	serious ^a^	very serious ^d^	not serious	very serious ^c^	none	⨁◯◯◯ Very low ^a,c,d^	SMD 0.54 SD lower (1.27 lower to 0.18 higher)
Craving Sub-score							
226 (3 RCTs)	serious ^a^	very serious ^d^	not serious	very serious ^c^	none	⨁◯◯◯ Very low ^a,c,d^	SMD 0.66 SD lower (1.51 lower to 0.18 higher)
Water Retention Sub-score							
96 (2 RCTs)	serious ^a^	Serious ^b^	not serious	very serious ^c^	none	⨁◯◯◯ Very low ^a,b,c^	SMD 0.37 SD lower (1.04 lower to 0.31 higher)

CI: confidence interval; SMD: standardized mean difference. Explanations: a. Most trials showed some concerns of bias. b. I^2^ > 50%. c. A wide confidence interval that does not exclude the appreciable harm or benefit, with a low number of participants. d. I^2^ > 75%.

## Data Availability

All data are available within the manuscript and its Supplemental Files.

## Supplementary Materials

The following supporting information can be downloaded at: https://www.mdpi.com/article/10.3390/jcm15124828/s1, Table S1: PRISMA checklist; Table S2: Search strategy; Table S3: Excluded records in full-text screening; Figure S1: Leave-one-out sensitivity analysis of total premenstrual syndrome score; Figure S2: Galbraith plot of total premenstrual syndrome score; Figure S3: Leave-one-out sensitivity analysis of depression sub-score; Figure S4: Leave-one-out sensitivity analysis of physical symptoms sub-score; Figure S5: Galbraith plot of depression sub-score; Figure S6: Galbraith plot of physical symptoms sub-score; Figure S7: Leave-one-out sensitivity analysis of anxiety sub-score; Figure S8: Galbraith plot of anxiety sub-score; Figure S9: Leave-one-out sensitivity analysis of craving sub-score; Figure S10: Galbraith plot of craving sub-score.
